# Sexual and Reproductive Health Behaviors Among Young Black Women in the U.S. Before the COVID-19 Pandemic: Insights for Addressing Future Challenges in STI and Pregnancy Prevention Within Key Female Populations

**DOI:** 10.3390/ijerph22050793

**Published:** 2025-05-17

**Authors:** Laurenia C. Mangum, Jaih Craddock

**Affiliations:** 1 Jane Addams College of Social Work, University of Illinois Chicago, Chicago, IL 60607, USA; 2 Social Justice and Ethics Forward Research Institute, Irvine, CA 92618, USA; jaihcraddock@gmail.com

**Keywords:** HIV prevention, reproductive/sexual health, Black women

## Abstract

New STI/HIV diagnoses disproportionately impact U.S. Black cisgender women at higher rates compared to other racial/ethnic U.S. cisgender women. Biomedical HIV prevention interventions, such as HIV Pre-Exposure Prophylaxis, have demonstrated lower uptake among Black women. Given the need to further develop prevention interventions that meet the sexual and reproductive health (SRH) needs of U.S. Black women, this study aimed to identify and characterize condom use behaviors, sexual communication efficacy, and SRH healthcare utilization among sexually experienced young Black women (YBW) aged 18–25 (N = 206). Participants completed a self-administered questionnaire assessing sexual and conception vulnerability, sexual/reproductive healthcare utilization, and sexual communication efficacy. Descriptive statistics and univariate analyses were conducted to identify correlations in sexual and reproductive behaviors. The results showed that 32% of YBW used a condom during their last sexual encounter. Five distinct trends in condom use were identified, three of which included condomless vaginal/oral sex. YBW reported high levels of sexual communication with sexual partners and consistent engagement in sexual/reproductive health care, including abortion care. Young Black women have diverse sexual/reproductive health needs and require culturally responsive patient-centered clinical practices aimed at reducing STI/HIV rates and unintended pregnancies. Future research could examine healthcare providers’ understanding of Black women’s SRH priorities and assess how this knowledge aligns with or diverges from established clinical guidelines and best practices. Such inquiries could illuminate potential gaps in provider education and clinical practice, ultimately informing the development of care models that are both evidence-based and responsive to the lived experiences of young Black women.

## 1. Introduction

Sexual and reproductive health (SRH) is a critical component of well-being for young women aged 18–25, a life stage marked by key developmental transitions and increased engagement in sexual exploration. This period often represents the first time many young women are navigating sexual relationships, which, if not supported by access to protective measures, may result in adverse health outcomes such as the acquisition of sexually transmitted infections (STIs), including HIV, or an unintended pregnancy among those with the capacity for conception [1]. Notably, the Centers for Disease Control and Prevention (CDC) reports that women aged 18–25 experience the highest rates of newly diagnosed STIs and HIV [2]. Given the intersection of heightened reproductive capacity and increased vulnerability to STIs, it is imperative that healthcare providers are equipped to respond to the SRH needs of this population. Provider responsiveness through culturally informed, developmentally appropriate care plays a vital role in reducing the disease burden, promoting health, and delaying unintended pregnancies among young women.

Among U.S. young Black women (YBW), SRH disparities are gravely poignant. Black American cisgender women disproportionately receive new Human Immunodeficiency Virus (HIV) diagnoses (92% via heterosexual contact) at higher rates than other racial/ethnic women, compared to their relative population representation in the United States (U.S.) [2]. Similarly, a 2023 Centers for Disease Control and Prevention STI Surveillance Report revealed that Black American cisgender women aged 15–24 are disproportionately diagnosed with STIs, including Chlamydia (15–19 years, 92,643 reported cases, 20–24 years 100,181 reported cases), Gonorrhea (15–19 years, 26,262 reported cases, 20–24 years, 29,498 reported cases), and Syphilis (15–19 years, 386 reported cases, 20–24 years, 872 reported cases) [3]. The disparities in STI diagnoses highlight the urgent need for targeted interventions and preventative measures that address the sexual health needs of Black American women, particularly in relation to STI screening and testing. Preventative sexual health service utilization measures, such as screening and testing for STIs, are essential, as having a recent bacterial STI is a risk factor for HIV acquisition [2]. Concerted efforts to address sexual health inequities among Black women include Healthy People 2030’s STI goal of reducing sexually transmitted infections and their complications, and improving access to quality STI care [4]. In addition, the national HIV/AIDS strategy, Ending the HIV Epidemic in the U.S. (EHE), seeks to reduce new HIV cases in the U.S. by 75% by 2025 and subsequently reduce new HIV cases by 90% by 2030 [5]. Building on these goals, increasing access to preventative measures like condoms and ensuring consistent use is crucial in reducing HIV transmission.

Reproductive health is often considered the comprehensive care concerning birth, including prenatal, perinatal and postpartum care, but also concerning the delaying of pregnancy to include family planning (contraception) and abortion care. Options to delay pregnancy within the family planning realm often include the pill, barrier methods such as condoms and diaphragms, long-acting reversible contraceptives like implants, the vaginal ring, IUD, sterilization and emergency contraception [6]. Among young Black women, access to and utilization of such contraception methods are multifactorial, including insurance status, the distance of the clinic or healthcare setting, individuals’ comfort in navigating use, and social policies that restrict, prohibit, or promote the use of contraception and its utility [7,8]. Regarding restrictions on reproductive health care choices and bodily autonomy, in 2022, the U.S. Supreme Court ruled on Dobbs v. Jackson Women’s Health Organization, which overturned Roe v. Wade, the landmark decision that had protected a woman’s constitutional right to access an abortion [9]. The Dobbs ruling returned the authority to regulate abortion to individual states, leading to significant variations in access to abortion services across the country. This decision has profound implications for sexual and reproductive health, particularly for marginalized groups, including Black women, who may already face barriers to healthcare access [10]. This ruling is relevant to the current research study as it highlights the intersection of healthcare access, reproductive rights, and social inequities. Given that Black women are disproportionately affected by systemic healthcare disparities, this decision underscores the need to examine how policies and access to care impact their sexual and reproductive health decisions, including condom use, contraception, and STI prevention. Understanding these factors is critical in designing effective interventions and advocating for equitable healthcare policies.

### 1.1. Biomedical HIV Prevention Interventions

The existing literature suggests that correct and consistent condom use (external and internal) is highly effective in preventing HIV and STI transmission during penetrative vaginal sex and receptive anal sex [11,12,13]. In addition, condoms are widely accessible, low-cost or free, do not require a prescription for use, have minimal side effects, and have single-use utility [14]. Furthermore, their effectiveness in preventing STI/HIV transmission, and demonstrated efficacy in preventing unintended pregnancy, thus demonstrate their viability of multipurpose prevention technologies for women [15]. Despite their advantages, challenges to condom use exist among sexual partners engaging in receptive anal sex or penetrative vaginal sex [16,17].

HIV Pre-Exposure Prophylaxis (PrEP) has been deemed successful at preventing HIV through sexual transmission when taken before exposure to HIV [18]. This biomedical prevention intervention provides women with the autonomy to maintain a layer of protection and control over their bodies. Full efficacy does require cisgender women to take the medication for 21 days for full maximum protection [19]. In the United States, Truvada, oral PrEP, has low uptake among Black women [20,21]. Dapivirine, similar to the contraceptive vaginal ring NuvaRing, is an approved PrEP method for cisgender women and has been used in sub-Saharan African countries to reduce cases of HIV [22]. Despite its success and approval by the European Medicines Agency, a review of Dapivirine ended with the U.S. FDA, in December 2021, leaving U.S. women with limited options for PrEP [23]. Despite that setback, cabotegravir long-acting injectable PrEP, taken every 8 weeks, was approved by the U.S. FDA in December 2021, becoming available for use among women [24].

While there have been advances in biomedical prevention interventions globally, research over the past decade has demonstrated a consensus that PrEP is little known and has low uptake among young Black women (YBW) [25,26]. Given the multilevel determinants of the low uptake of PrEP, it is critical that HIV prevention researchers continue examining patterns of sexual behavior among Black women to understand standing and emerging barriers to condom use and to address the continuous sexual health disparities.

### 1.2. Sexual Communication Self-Efficacy

Sexual communication self-efficacy is one factor that is known to influence sexual health behavior, such as condom use [27]. Previous studies have recognized that low condom negotiation self-efficacy and low sexual communication self-efficacy can constrain condom use discussions among sexual partners [28,29,30]. A study by Javier and colleagues (2018) concluded that increased confidence in communicating about sexual situations with a partner and higher scores of condom negotiation efficacy were associated with consistent condom use among Black American women [31]. Like sexual communication self-efficacy, gender differences and relational power within heterosexual relationships can impact condom use discussions, the perceived intent to use condoms, and consistent condom use among racially minoritized women [32]. Namely, unhealthy power differences and intimate partner violence can impact coercive condom non-use, stealthing, which is nonconsensual condom removal, and condom sabotage, which is destructing the condom [33]. On the contrary, some authors have also suggested that greater dyadic trust in exclusive dating relationships is a contributing factor to increased sexual communication efficacy and consistent condom use among college women [34].

While there is a substantial body of research on consistent condom use and condom use self-efficacy among Black American cisgender women in heterosexual relationships, studies examining condom use by type of sexual encounter (e.g., oral, anal, vaginal sex) among young Black women are limited. This study fills a critical gap in what is known regarding condom use behaviors by sexual activity type, in addition to providing insights regarding SRH care engagement among a sample of YBW. The purpose of this study was to identify and characterize sexual behaviors, partner communication, and SRH care involvement among sexually experienced YBW.

## 2. Materials and Methods

### 2.1. Data Sources

Data for this secondary data analysis study come from the Sex, Relationship, and Health Study [35]. Data were collected from June 2018 through to December 2018. Individual-level data and social network-level data were collected from participants via a self-administered electronic survey. The individual-level data captured responses within the topic areas of HIV/STI risk behaviors, sexual/reproductive health, relationship dynamics, sexual discussions, HIV PrEP knowledge, attitudes, and use, including oral and vaginal PrEP, and health-related social media use [35]. Thus far, studies from this data include findings on oral PrEP interest and preventative sexual health service utilization [26], findings on oral PrEP vs. vaginal PrEP interest [27], social network characteristics and individual-level vulnerabilities, and PrEP interest [36]. A full description of the methods used in the original study is outlined by Craddock (2019). This study utilizes individual-level data.

### 2.2. Study Population

The study sample consisted of 209 YBW aged 18–25, residing throughout the United States. The inclusion criteria for YBW in this study were being sexually active at the time of the study (N = 184). Being sexually active was defined as having vaginal, anal, or oral sex within the past 6–12 months.

### 2.3. Measures

#### Demographics

Participant *age* was assessed categorically: “How old are you now [18, 19, 20, 21, 22, 23, 24, 25, or 26 or older]?” Education level was assessed categorical: “What is the highest grade in school or year of college that you have completed and got credit for? (A GED would be 12 years)” (e.g., less than high school graduate … 4-year degrees). The demographic screening question for health insurance was captured with the following question: “Have you had health insurance in the past year [yes = 1 or no = 0]?” Relationship status was assessed categorically: “What is your current relationship status (select all that applies)?” (e.g., single, dating, friends with benefits or casual sex partner, committed relationship). Geographical regions of residence were assessed nominally, with participants providing their six-digit residency zip code. Data were then collapsed into corresponding U.S. Census geographic regions: Northeast, Midwest, South, and West [37].

### 2.4. Variables

#### 2.4.1. Sexual Health Behaviors and Healthcare Utilization

Time since last sexual encounter was captured with the following question: When was the last time you had sex (oral, vaginal, or anal)? (e.g., within the last week … one year or longer). Last sexual partner was captured with the following: “Think about the last person you had sex (oral, vaginal, or anal) with. How would you describe this partner? (e.g., life partner, boyfriend or girlfriend, hookup, or casual sex partner)”. Frequency of sexual intimacy was captured with the following: “How long have you been having sex with this partner?” (e.g., 1 time, less than 1 week … 6 months and 1 year, 1 year or longer). Types of last sex and condom use were assessed categorically: “The last time you had sex, what kinds of sex did you have? Check all that apply (e.g., anal sex with a condom, vaginal sex, no condom)”. STI testing was captured with the following question: “Have you ever been tested for a sexually transmitted infection, or STI or STD, for example, Chlamydia, gonorrhea, syphilis, genital warts?” (yes or no). Recent STI testing was assessed with the following question: “When was the last time you were tested for sexually transmitted infection, or STI or STD, for example, Chlamydia, gonorrhea, syphilis, genital warts? (e.g., within the past 3 months … 6 or more months ago)”. HIV testing was captured with the following: “Have you ever been tested for HIV?”. Recent HIV/AIDS testing was assessed categorically: “When was the last time you were tested for HIV/AIDS? (e.g., within the past 3 months … 6 or more months ago)”. Recent Pap test was measured using one item: “(in the past 12 months, have you received) A Pap test, where a doctor or nurse put an instrument in the vagina and took a sample to check for abnormal cells that could turn into cervical cancer?” (yes or no).

#### 2.4.2. Sexual Communication Efficacy

Recent sex partners was assessed categorically: “It is hard to ask someone if they have had sex with more than one person in the last year”. Responses were rated on a 4-point Likert scale ranging from strongly agree to strongly disagree. For this analysis, strongly agree and agree responses were coded as 1 and disagree and strongly disagree responses were coded as no = 0. Prior Sex Partners was assessed categorically: “I am able to ask my sex partner how many people they have had sex with before me”. Sexual Partner Anal Sex Experiences was assessed categorically: “I am able to ask my sex partner(s) if they have ever had anal sex.” Responses ranged from strongly agree to strongly disagree. For this analysis, strongly agree and agree responses were coded as 1 and disagree and strongly disagree responses were coded as no = 0. Sexual Partner IV drug use was assessed categorically: “I am able to ask my sex partner(s) if they have ever used IV drugs”. Corresponding responses ranged on a 4-point Likert scale ranging from strongly agree to strongly disagree. For this analysis, strongly agree and agree responses were coded as 1 and disagree and strongly disagree responses were coded as no = 0. Male to Male Sexual Contact was assessed categorically: “I am able to ask my male sex partner(s) if they have ever had sex with another man”. Corresponding responses ranged on a 4-point Likert scale ranging from strongly agree to strongly disagree. For this analysis, strongly agree and agree responses were coded as 1 and disagree and strongly disagree responses were coded as no = 0.

Partner condom use was assessed using a single item: “I am able to discuss the use of condoms with my sex partner.” Corresponding responses ranged on a 4-point Likert scale from strongly agree to strongly disagree. Lastly, power in the relationship was assessed categorically: “In general, who do you think has more power in your relationship?” Corresponding responses were as follows: I do, my partner does, we both do equally, neither of us does, and unsure.

#### 2.4.3. Reproductive Health Behaviors and Care Utilization

Pregnancy prevention was captured with the following question: “The last time you had vaginal sex, what method(s) did you or your partner use to prevent pregnancy? Check all that apply”. Contraception use was captured with the following question: “In the past 12 months, have you received a method of birth control or a prescription for a method?” Emergency contraceptive use was captured with the following question: “In the past 12 months have you received emergency contraception, also known as “Plan B” or the “Morning-after pill” or a prescription for it?” Recent pregnancy testing was captured with the following question: “You may have already told me, but in the past 12 months, have you received a pregnancy test?” Utilization of abortion care services was captured with the following question: “In the past 12 months, have you received an abortion?”

### 2.5. Data Analysis Plan

Univariate analyses, including measures of central tendency, measures of variability, and frequency statistics, were conducted on demographics, SRH behaviors, healthcare utilization, and sexual communication efficacy. We conducted all analyses using Stata 18 software [38]. The Institutional Review Board approved this study at the University of Southern California.

## 3. Results

### 3.1. Socio-Demographics

Participant demographics and characteristics are presented in Table 1.

### 3.2. Sexual Behaviors and Healthcare Utilization

Among sexually experienced YBW (N = 184), 45% reported that their last sexual encounter was with a boyfriend or girlfriend, and 35% of YBW had been sexually intimate with that partner for a year or longer. Approximately one-third (32%) of YBW reported having sex (anal, oral, or vaginal) within the last week. Among the sample, 43% reported receiving a PaP (Smear) test within the last year. Over two-thirds (66%) reported ever being tested for an STI, and (66%) reported ever being tested for HIV/AIDS. Lastly, 63% of YBW reported receiving an STI test within the last six months, while 57% of YBW reported receiving an HIV test within the last six months to a year.

**Figure 1:**
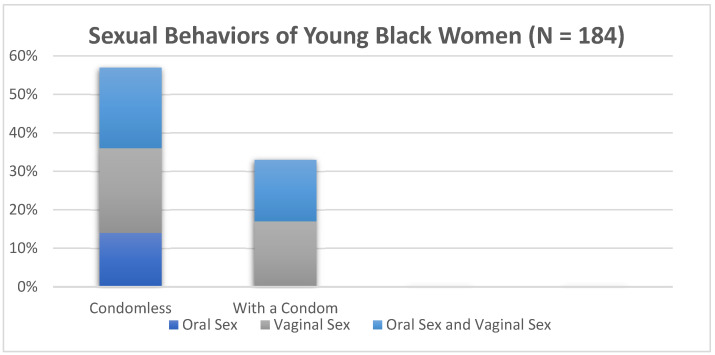
Sexual behaviors of young Black women (N = 184).

Table A1 of Appendix A provides an overview of the sexual and reproductive health behaviors of the sample. Young Black women reported their condom use behaviors in tandem with the corresponding types of sex they had with their last sex partner. Figure 1 provides a breakdown of the condom use trends. Of YBW (N = 184), 14% reported their last type of sex as condomless oral sex, while 21% of YBW reported their last type of sex as a combination of condomless oral sex and condomless vaginal sex. Additionally, 22% of YBW reported having condomless vaginal sex. Sixteen percent of YBW reported their last type of sex as a combination of condomless oral sex and protected vaginal sex with a condom. In contrast, 17% of YBW reported their last type of sex as protected vaginal sex with a condom.

### 3.3. Sexual Communication Efficacy

Of YBW, 40% reported that, in general, both partners have equal power within the relationship. In terms of sexual communication, 72% of YBW reported ease in asking their sex partner if they had sex with more than one person in the last year, while 85% of YBW reported that they were able to ask their sex partner the total number of people they had sex with prior to them. Ninety-seven percent of YBW reported being able to discuss condom use with their sex partners. Eighty-six percent of YBW reported being comfortable asking their male sex partner about their history of anal sex, 86% felt comfortable discussing injection drug use with their sex partners, and 71% were comfortable asking their male sex partner if they have ever had sex with another man.

### 3.4. Reproductive Health Behaviors and Healthcare Utilization

**Figure 2:**
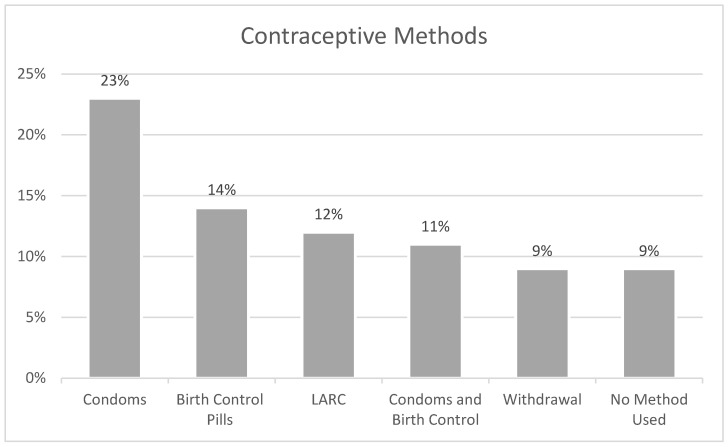
Contraceptive methods most commonly used among young Black women (N = 183).

Of YBW who reported engaging in reproductive health behaviors and healthcare utilization in the past 12 months (N = 183), over half of the sample (57%) reported receiving a method of birth control or a prescription for a method, while 50% of YBW received counseling about a method of birth control. Along those lines, 47% of YBW received a check-up in the past year for medical tests relating to using a birth control method. In terms of contraception use, 23% of YBW reported using condoms, 14% reported using birth control pills, while 12% of YBW reported using a long-acting reversible contraception method such as Depo-Provera (or any injectable birth control), NuvaRing (or any birth control ring), Implanon (or any implant), or any IUD (Figure 2). In addition, 11% of YBW used a combination of condoms and birth control pills, 9% used pulling out/withdrawal, and another 9% reported not using any method to prevent pregnancy. In the past 12 months, 15% of YBW reported receiving emergency contraception such as “Plan B” or the “Morning after pill,” one-third of YBW (30%) reported receiving a pregnancy test, and 3% of YBW reported terminating a pregnancy via an abortion.

## 4. Discussion

This study aimed to identify and characterize condom use behaviors, sexual communication efficacy, and SRH care utilization among sexually experienced young Black women (YBW) aged 18–25. The findings provide valuable insights into factors that may influence health outcomes in this population. A summary of the findings situated within existing research and implications for policy, research, and population health are presented below.

### 4.1. Sexual Health Behaviors and Sexual Healthcare Utilization

In this study, a marginal proportion of sexually experienced YBW (33%) reported using condoms during their last sexual encounter. These findings were lower than similar studies that found a higher percentage of YBW used condoms during their last sexual encounters (41.1%; 47%, respectively) [39,40]. One potential explanation for low condom usage among this sample may be due to the low perceived risk of STI and HIV acquisition [39,40], as they may believe that they are in a committed, monogamous relationship, absent of sexual concurrency. A substantial proportion of the sample (45%) reported that their last sexual encounter was with a girlfriend or boyfriend (with 6% being same-sex relationships and 94% being heterosexual relationships), and of those YBW, over one-third (35%) had had sexual relationships for a year or longer with that partner. This is pertinent to discussions of condom use within sexual relationships, as individuals’ decisions regarding condom use may vary based on the nature of the relationship. For instance, consistent condom use may be more likely when a partner is perceived to engage in sexual activity with multiple individuals, in contrast to scenarios involving monogamous relationships, where perceived exclusivity may reduce the perceived need for protection. In similar studies of condom use among YBW, monogamy was a common factor for the lack of condom use [39,40]. This can also be connected to the trust a woman has in her partner and the U.S. societal belief that being in a monogamous relationship reduces the risk of HIV, even among YBW who have more knowledge and information about HIV and HIV prevention [41,42]. Along the lines of trust in a relationship, YBW who are in heterosexual relationships may rely on a male sex partner to provide external condoms during sexual intercourse. While internal condoms are effective, they often are not feasible during intercourse due to their bulky design and women’s lack of confidence in using them [43]. As such, women may abdicate their power and bodily autonomy to their male sexual partner to use an external condom [44]. Additionally, some YBW reported mixed condom use within a single sexual encounter. YBW reported engaging in condomless oral sex followed by vaginal intercourse with a condom. These findings underscore the nuanced and context-dependent nature of condom use, illustrating how both protected and unprotected sex can occur within the same encounter. Notably, some YBW initiated sex with a condom but subsequently removed it, reflecting fluid decision-making processes and the dynamic interplay of factors influencing condom use. This pattern of mixed condom use remains underexamined in the literature. Yet, it is critical to understanding the barriers to consistent condom use and how these behaviors may evolve over time. Such moments may serve as strategic inflection points for the introduction of HIV pre-exposure prophylaxis (PrEP) as a complementary HIV prevention strategy. Offering daily oral or long-acting injectable PrEP options may provide YBW with greater autonomy and protection against HIV acquisition in situations where condom use is inconsistent.

Another potential explanation for the underutilization of condoms could be that sexual arousal, impulsivity, and attitudes towards condom use may be impacting condom use decision-making among young Black men in heterosexual relationships with YBW. A study examining young men’s condom resistance tactics concluded that men fell into three distinct groups based on attitudes toward women, sexual sensation seeking, and condom use beliefs, suggesting that condom use decision-making extends beyond sexual impulsivity [45,46]. Similarly, decisions regarding when and how to use condoms in a relationship may be influenced by YBW’s desire not to disrupt their partner’s sexual arousal and sensation; this may result in Black women experiencing unmet SRH needs [46]. To explicate further, Crooks and colleagues’ study of the sexual development of Black women concluded that Black women acquiesce to their partners during sexual intercourse as a way to protect Black men and affirm them in the sacred space of intimacy [47]. This further underscores the importance of incorporating discussions of PrEP use into clinical encounters to ensure that the sexual health needs of YBW are adequately addressed. Moreover, engaging both sexual partners in conversations about PrEP may also help challenge prevailing misconceptions, particularly the notion that PrEP is primarily intended for queer men who have sex with men. Initiating PrEP prior to sexual activity or opting for a long-acting injectable PrEP, which offers protection for up to eight weeks, may serve as effective strategies for mitigating the risk of HIV acquisition, especially in instances of unplanned or spontaneous condomless sex.

### 4.2. Sexual Communication Efficacy

Despite our findings on low condom usage, our study demonstrated that YBW have an enhanced capacity for sexual communication with their partners. This finding was similar to other studies examining sexual communication among young Black college-educated women [46]. This may be the case because YBW have entered a period of sexual development where they perceive having a greater sense of themselves and are active participants in their sexuality and sexual experiences [48]. Higher rates of sexual communication among YBW could also be a result of more inclusive and tolerant sexual diversity, more broadly among Generation Z. The sample was mostly college students who have been exposed to diverse people with various gender identities and expression, and thus are destigmatized to behaviors that are traditionally considered private and taboo topics for Black women to discuss. This could also drive the perceived equal power amongst both partners in their relationships. In addition, many participants were from the western region of the United States (California), which tends to be more liberal and accepting of gender diversity and sexual identity [48]. The ability to engage in open and honest conversations about sexual health presents a valuable opportunity to introduce discussions about PrEP within peer networks, potentially increasing awareness and acceptance of its efficacy among YBW. Additional research is warranted to explore how communication around condom use has evolved among YBW across generational cohorts, including Generation X (individuals born between the mid-1960s and late 1970s), Millennials (born between the early 1980s and mid-1990s), and Generation Z (born between the mid-1990s and early 2000s). Investigating these generational shifts is critical, as one’s SRH socialization may significantly shape attitudes, beliefs, and behaviors related to SRH practices. Such studies should also consider how older generations receive and interpret messaging about PrEP, including awareness and perceptions of various PrEP modalities.

### 4.3. Reproductive Health Behaviors and Healthcare Utilization

Our study found that a substantial number of YBW (57%) were aware of contraceptive methods and were using them. These findings suggest a relatively high level of engagement with healthcare services related to birth control, which is consistent with the literature on the importance of counseling and education for contraceptive use [49]. However, only 23% of YBW reported using condoms as a contraceptive method, 14% reportedly used pills, and 12% reported using long-acting reversible contraception (LARC), indicating that while contraceptive use is common, there is room for improvement in promoting condom use as part of a dual protection strategy to prevent both pregnancy and STIs. In fact, condoms were the most commonly endorsed method of contraception. These findings are similar to studies examining contraceptive preferences among Black women, which found that Black women preferred to use contraceptive methods that were easy to control and manipulate without requiring a provider and that provide protection against STIs during intercourse [50]. The fact that U.S. Black women want bodily autonomy in the type of contraception may be a result of the structural racism experienced in the past and the reproductive harm experienced by healthcare providers [51]. Furthermore, condoms, as a primary barrier method of contraception, offer the advantage of being readily accessible and do not require ongoing engagement with healthcare providers. This is particularly significant for uninsured or underinsured individuals, for whom access to healthcare services may be limited.

The reproductive health behaviors reported in this study also reveal several trends that warrant further attention and investigation. While 66% of YBW reported ever being tested for an STI and 66% reported ever being tested for HIV, only 43% received a Pap smear within the past year, which suggests that regular screenings and preventive care may not be as universally prioritized. This is consistent with existing research, which found that young women, particularly those from marginalized communities, may face barriers to accessing routine preventive services, such as Pap smears and STI testing, despite having health insurance [51]. This may also reflect the evolving clinical guidelines recommending that patients undergo Pap testing every two to three years, rather than annually, for cervical cancer screenings [52].

Finally, reproductive health behaviors related to emergency contraception use and pregnancy testing offer important contextual insight into broader sexual health practices and decision-making. Approximately 15% of YBW reported using emergency contraception in the past year, and 30% had a pregnancy test. These findings may reflect both proactive health behaviors and a need for improved access to contraceptive methods to prevent unintended pregnancies. This may also indicate a broader need for comprehensive menstrual health education, including fertility awareness, ovulation tracking, and strategies to better understand reproductive cycles, all of which can offer additional support in preventing unintended pregnancies. Furthermore, these findings elucidate the importance of advancing multipurpose prevention technologies (MPTs) in the United States, which simultaneously offer protection against both HIV and unintended pregnancy, addressing multiple sexual and reproductive health needs in a single intervention.

### 4.4. Implications for Research, Policy, and Clinical Practice and Education

The findings of this study highlight the need for further research into the factors that influence condom use and contraceptive choices among YBW. While many YBW engage in open communication with their partners about sexual health, there is still a significant proportion of YBW that do not use protection consistently. Understanding the barriers to condom use, as well as the factors that lead to the adoption of certain contraceptive methods, is crucial for designing targeted interventions. This is particularly important as PrEP uptake remains low among Black cisgender women for a multitude of reasons, including a lack of awareness, low HIV risk perception, a lack of providers prescribing it, and medication costs. Longitudinal research could help determine the factors that influence the adoption of safer sexual practices over time and the long-term effectiveness of interventions. Of course, such things as family planning and reproductive age may impact condom use decision-making. Therefore, conducting this research among adolescents and emergent adults may be more fruitful. It would be helpful to determine the societal influences on their decision-making and sexual socialization. If we have more data in this area, we can better develop interventions to address their SRH needs. Additionally, exploring how reproductive health behaviors, such as contraceptive use and emergency contraception, are related to HIV and STI prevention would provide valuable insights into comprehensive sexual health strategies. The development of HIV prevention interventions should take into consideration the idiosyncrasies of Black women, and, more specifically, regional differences in approaches to sexual and reproductive health. Future studies should explore types of event-based sex, the types of sex, and the frequency of sex to determine if there are unobservable groups among young Black women as it pertains to the environment where sex occurs (random guys, steady partners, social events, every so often). Similarly, we need to foster the many opportunities to educate young Black women about the benefits of PrEP while also providing harm reduction counseling to lower their risk of STI/HIV infection. PrEP use is ideal as it promotes autonomy among individuals who are unaware of their partners’ status, particularly for individuals experiencing sexual violence, coercion, and condom sabotage.

Policy initiatives should prioritize expanding YBW’s access to SRH services. This includes improving access to a range of contraceptive methods, from condoms to long-acting PrEP options to long-acting reversible contraception (LARC), as well as ensuring that services are culturally sensitive and tailored to the needs of young women in marginalized communities. Policies should also focus on increasing the availability of reproductive health counseling and information on contraception, menstruation, and HIV/STI prevention. It is important to have conversations about SRH in tandem, as having an STI can impact future fertility and pregnancy. Furthermore, social policies should promote comprehensive sexual health education that covers both contraception and safer sex practices, addressing the social and cultural factors that influence sexual decision-making as opposed to abstinence-only curricula. Importantly, policies aimed at reducing the stigma surrounding abortion, emergency contraception, and HIV testing will help YBW seek the care they need without fear of judgment. Given the current restrictions on reproductive justice, such as abortion care and the impending restrictions on contraception use in the United States at the federal level, support at the state level and from community-based organizations is greatly needed.

In clinical practice, our findings can support revisions to the clinical guides and assessments used when providing sexual preventative health care to this population. For example, sexual history assessments can be modified to include additional probing questions about condom use (e.g., how many times during that sexual encounter do you use a condom, do you keep the condom on the entire time, does the condom go on and is it removed later on). They should also include questions about PrEP awareness and interest. It is important to include conversations about both options, as there are differing reasons for wanting to use condoms vs. PrEP, and historical and sociopolitical factors also influence one’s decision-making regarding biomedical prevention interventions.

Healthcare providers should integrate routine discussions of sexual and reproductive health (SRH) into standard care for YBW. It is equally important that clinical environments foster open, nonjudgmental communication, empowering YBW to voice their sexual health concerns and inquire about potential risk factors or warning signs in their relationships. To ensure these interactions are both effective and respectful, providers must be trained in culturally competent care that acknowledges and responds to the specific social and cultural contexts affecting YBW. Moreover, providers should demonstrate a clear and professional comfort in discussing a range of sexual practices—including oral-anal contact (rimming), oral–vulvar contact (cunnilingus), and oral–penile contact (fellatio)—and offer tailored strategies for the prevention of sexually transmitted infections (STIs), HIV, and unintended pregnancy. Educational initiatives should focus on both SRH, providing young Black women with the knowledge and resources they need to make informed decisions about their health, ensuring that information is accessible and empowering. Programs should also emphasize communication skills, encouraging young women to discuss sexual health topics with their partners. Furthermore, education about reproductive rights and options, including emergency contraception and abortion, should be a part of these efforts to ensure that young women understand all the resources available to them.

### 4.5. Limitations

Overall, this study extends our knowledge of current SRH behaviors, healthcare utilization, and sexual communication among a representative sample of sexually experienced young Black women aged 18–25. It provides a roadmap of priority areas to consider when developing culturally relevant interventions to address STI/HIV prevention and unintended pregnancy. This dataset represents one of the most comprehensive sources of data on sexually experienced young Black women in the U.S. and remains invaluable for understanding their SRH behaviors. A limitation of the study is that social desirability bias may have impacted the response items of the survey due to the topic of sexual intimacy, sexual/reproductive health, and STI/HIV. It is possible that respondents selected response options that were more socially acceptable, considering how these topics are highly stigmatized among young Black women. Another limitation of the study is that the data were collected in 2018. The data were collected prior to the COVID-19 pandemic, and thus, they offer a pre-pandemic snapshot of SRH behaviors among YBW, unimpacted by subsequent changes in healthcare access and sexual behavior patterns. Much of the SRH landscape has changed globally in the last several years, with new advances addressing gaps in the SRH needs of Black women. However, in the United States, the findings remain relevant as disparities in SRH outcomes for Black women continue to persist. We also do not believe that the social phenomena of condom use behaviors in young Black women have changed since the data were initially collected in 2018. To this end, more recent data on this social phenomenon are not available.

## 5. Conclusions

Sexual and reproductive health behaviors and healthcare utilization among young Black women are nuanced, and their implications reach far beyond surface-level medical assessments and evaluations. They are often influenced by socio-political and cultural antecedents. As such, the sexual and reproductive behaviors of young Black women require targeted, culturally tailored, and gender-specific HIV prevention messaging to reduce STI acquisition and secondary HIV transmission. Interventions need to build upon Black women’s sexual development and equip them with the tools to engage in pleasurable and safer sex to reduce the community spread of HIV.

## Figures and Tables

**Table 1:** Participant demographics and characteristics (N = 206).

Characteristics		
	M (SD)	
Age	21.14 (1.74)	
	N	%
Education Level		
High School Graduate	28	13.40
Some College	92	44.02
Associate’s Degree	7	3.35
Bachelor’s Degree	73	34.93
Professional Degree	9	4.31
Current Health Insurance		
No	5	2.48
Yes	197	97.52
Relationship Status		
Single	90	42.86
Dating	20	9.52
Committed Relationship	53	25.24
Casual Sex Partner	9	4.29
Last Sexual Encounter		
Within the last week	60	32.43
1–4 weeks ago	39	21.08
1–3 months ago	44	23.78
3–6 months ago	17	9.19
6–12 months ago	13	7.03
1 year or longer	12	6.49
Last Sexual Encounter Partner Type		
Life Partner, Husband, Wife, Spouse	11	5.98
Girlfriend or Boyfriend	82	44.57
Hookup or Casual Sex Partner	53	28.8
Friends with Benefit	38	20.65

## Data Availability

The data that support the findings of this study are not publicly available due to their containing information that could compromise the privacy of research participants.

## Appendix A

**Table A1:** Sexual and reproductive health utilization among young Black women (N = 206).

Characteristics	N	%
Ever tested for HIV/AIDS		
No	70	33.98
Yes	136	66.02
Last time tested for HIV/AIDS		
Less than 6 Months	76	57.14
More than 6 months ago	57	42.86
Ever tested for STIs		
No	70	33.98
Yes	136	66.02
Last time tested for STIs		
Less than 6 months ago	85	62.96
More than 6 months ago	50	37.04
Pap (Smear) Test		
No	118	57.28
Yes	88	42.72
Birth Control Prescription in the past year		
No	89	43.2
Yes	117	56.8
Birth control related medical exam in the past year		
No	110	53.4
Yes	96	46.60
Birth Control Counseling in the past year		
No	103	50
Yes	103	50
Emergency Contraception Use in the past year		
No	176	85.44
Yes	30	14.56
Pregnancy Test in the past year		
No	144	69.9
Yes	62	30.1
Abortion Care in the past year		
No	198	96.59
Yes	7	3.41
